# Metformin Use Is Not Associated With B_12_ Deficiency or Neuropathy in Patients With Type 2 Diabetes Mellitus in Qatar

**DOI:** 10.3389/fendo.2018.00248

**Published:** 2018-05-25

**Authors:** Tarik Elhadd, Georgios Ponirakis, Zeinab Dabbous, Mashhood Siddique, Subitha Chinnaiyan, Rayaz A. Malik

**Affiliations:** ^1^National Diabetes & Endocrine Centre, Al-Wakra Hospital, Hamad Medical Corporation, Doha, Qatar; ^2^National Diabetes & Endocrine Centre, Hamad General Hospital, Hamad Medical Corporation, Doha, Qatar; ^3^Weill Cornell Medicine-Qatar, Qatar Foundation, Education City, Doha, Qatar

**Keywords:** metformin, vitamin B12 deficiency, diabetic neuropathy, diabetic painful neuropathy, type 2 diabetes mellitus

## Abstract

**Background:**

Metformin may lead to B_12_ deficiency and neuropathy. There are no published data on the prevalence of Metformin-related B_12_ deficiency and neuropathy in the Arabian Gulf.

**Aims:**

Determine whether Metformin intake is associated with B_12_ deficiency and whether B_12_ deficiency is associated with diabetic peripheral neuropathy (DPN) and painful diabetic neuropathy.

**Methods:**

Patients with type 2 diabetes mellitus (T2DM) (*n* = 362) attending outpatient clinics at HMC underwent assessment of B_12_ levels, the DN4 questionnaire, and vibration perception threshold (VPT).

**Results:**

Comparing Metformin to non-Metformin users there were no differences in B_12_ levels, VPT, or DN4. The prevalence of B_12_ deficiency (B_12_ <133 pmol/l) was lower (*P* < 0.01) in Metformin (8%) compared to non-Metformin (19%) users. Patients with B_12_ deficiency had a comparable prevalence and severity of sensory neuropathy and painful neuropathy to patients without B_12_ deficiency.

**Conclusion:**

Serum B_12_ levels were comparable between Metformin and non-Metformin users with T2DM in Qatar. T2DM patients on Metformin had a lower prevalence of B_12_ deficiency. Furthermore, the prevalence and severity of neuropathy and painful diabetic neuropathy were comparable between patients with and without B_12_ deficiency.

## Introduction

Metformin remains first-line therapy in type 2 diabetes mellitus (T2DM), with around 120 million users worldwide. It is increasingly used in overweight T2DM patients and those with polycystic ovary syndrome (1). Most international guidelines recommend Metformin after lifestyle measures for T2DM patients.

Metformin therapy was shown to be associated with a significant reduction in the level of vitamin B_12_ over 50 years ago (2, 3). A number of observational and placebo-controlled studies have confirmed that Metformin may reduce vitamin B_12_ levels (4–10). Indeed a recent study from Pakistan found that 29.7% of patients on Metformin had B_12_ deficiency (11) and another study from Brazil showed that B_12_ deficiency occurred in 22.4% of patients with T2DM on Metformin, and was further reduced in those on proton pump inhibitors (PPI)/H2-antagonists (10). However, a recent meta-analysis showed that only 10/17 studies showed that Metformin use led to B_12_ deficiency and in four prospective studies B_12_ was reduced by approximately 57 pmol/L, within 6 weeks to 3 months of commencing Metformin (12).

A potential consequence of B_12_ deficiency is that it could directly result in neuropathy or exacerbate diabetic neuropathy. Indeed, the recent 2017 ADA position statement on diabetic neuropathy has emphasized the importance of excluding B_12_ deficiency in patients with diabetic neuropathy (13). However, there are conflicting reports on the association between Metformin-induced B_12_ deficiency and neuropathy, with some reports showing an association (14, 15) while others have refuted this (11, 16–18). Furthermore, in a recent study from Turkey, while the prevalence of B_12_ deficiency was 38.4% there was no difference in B_12_ levels in those with and without neuropathy (19). Despite this there is wide spread administration of vitamin B_12_ therapy in patients in the Middle East and Far East, with a recent analysis from five teaching hospitals in Jordan, indicating that cyanocobalamin (B_12_), was the second most common injectable therapy after insulin (20). There are no published data on Metformin-related B_12_ deficiency or the relationship between B_12_ deficiency and diabetic neuropathy in the MENA region.

We have compared vitamin B_12_ levels in outpatients with T2DM in Qatar, in relation to Metformin use and further assessed for the prevalence and severity of painful neuropathy and sensory neuropathy in patients with B_12_ deficiency.

## Materials and Methods

Participants with T2DM (*n* = 362) were recruited from the National Diabetes & Endocrine Centers in Al-Wakra Hospital and Hamad General Hospital. The study was performed between March 6, 2017 and September 28, 2017.

Exclusion criteria included patients with a prior history of pernicious anemia, chronic kidney disease, previous bariatric surgery, gastrectomy, or small bowel resection for inflammatory bowel disease. This study was approved by the Institutional Review Board (IRB) of WCM-Q and HMC and all participants gave informed consent to take part in the study. The research adhered to the tenets of the declaration of Helsinki.

### Demographic and Blood Measures

Data including age, duration of diabetes, blood pressure, body mass index (BMI), and medications including Metformin were recorded. HbA1c, lipid profile, renal function, and serum B_12_ were assessed.

### B_12_ Assay

Blood was drawn directly into a dedicated evacuated tube (BD Diagnostic—Preanalytical Systems, Oxford, UK) and centrifuged at 3,500 *g* for 10 min and serum analyzed immediately or stored at −20°C until analysis on Beckman Dxi 600 (Beckman Coulter Inc., Brea, CA, USA). The Vitamin B_12_ assay is a competitive-binding immunoenzymatic assay. The amount of analyte in the sample was determined by means of a stored, multipoint calibration curve (Beckman Coulter Assay Manual 2015, Beckman Coulter Inc., Brea, CA, USA). Analytical sensitivity <50 pg/ml, traceability; traceable to an internal standard manufactured using the purified cyanocobalamin. Assay precision: 4.8–11.4%. B_12_ levels <133 pmol/l were considered deficient.

### Diabetic Peripheral Neuropathy (DPN) Assessment

Vibration perception threshold (VPT) was measured on the pulp of the large toe with a Neurothesiometer (Horwell, Scientific Laboratory Supplies, Wilford, Nottingham, UK). The test was repeated three times and the average value was recorded. VPT at a cutoff point ≥15V was defined as DPN (21).

### Neuropathic Pain Assessment

The Douleur Neuropathique 4 (DN4) was used to identify neuropathic pain (22, 23). The DN4 is comprised of 10 questions (7 symptoms and 3 signs) and a score ≥4 identifies neuropathic pain with high sensitivity (83%) and specificity (90%) (24).

### Statistical Analysis

Variables were compared between groups using a *t*-test and χ^2^ test for continuous and categorical data, respectively. Data are expressed as mean (SD) of mean.

Univariate analysis by simple linear regression was applied to determine which variables are associated with B_12_ levels, VPT, and DN4 as outcome measures. Multiple linear regression analysis was used to determine the association between B_12_ levels, VPT, and DN4 after adjusting for confounding factors. Assumptions of linear regression were satisfied for normality, collinearity, and outliers. Additionally, residual plots were used to determine whether the models fit the assumptions.

All analyses were performed using StatsDirect version 3.0. A two-tailed *P* value of <0.05 was considered significant.

## Results

Age, systolic (SBP), BMI, HbA1c, triglycerides, HDL, and B_12_ levels were comparable between Metformin (*n* = 235) and non-Metformin users (*n* = 64). Metformin users had a shorter duration of diabetes (10.27 ± 7.45 vs 12.89 ± 8.89, *P* = 0.03), but higher diastolic blood pressure (DBP) (77.72 ± 9.91 vs 74.52 ± 9.42, *P* = 0.02), total cholesterol (4.48 ± 1.10 vs 4.15 ± 1.02, *P* = 0.03), and LDL (2.56 ± 0.88 vs 2.30 ± 0.82, *P* = 0.04). B_12_ levels were comparable between Metformin and non-Metformin users (*P* = 0.87). However, the prevalence of B_12_ deficiency was lower in Metformin (8%) compared to non-Metformin (19%) users, *P* < 0.01.

The prevalence of neuropathy (30 vs 39%) and neuropathic pain (31 vs 33%) were comparable between Metformin and non-Metformin users. The proportion of patients taking medications, which could influence B_12_ levels, including PPI, calcium supplements, multivitamins, B_12_ supplements, and sulfonylureas were comparable between Metformin and non-Metformin users (Table 1).

**Table 1:** Comparison of demographic and clinical characteristics between non-Metformin users and Metformin users.

	Non-metformin users (*n* = 64)		Metformin users (*n* = 235)		*P* value
**Demographics**					

Age (years)	52.67	(13.95)	54.19	(11.61)	0.43
Diabetes duration (years)	12.89	(8.89)	10.27	(7.45)	0.03
SBP (mmHg)	128.88	(18.37)	130.23	(18.72)	0.61
DBP (mmHg)	74.52	(9.42)	77.72	(9.91)	0.02
BMI (kg/m^2^)	31.79	(7.47)	32.10	(7.70)	0.78
HbA1c (%)	8.41	(2.22)	7.86	(1.89)	0.07
Total cholesterol (mmol/l)	4.15	(1.02)	4.48	(1.10)	0.03
Triglycerides (mmol/l)	1.67	(1.12)	1.82	(1.14)	0.35
HDL (mmol/l)	1.15	(0.52)	1.05	(0.29)	0.18
LDL (mmol/l)	2.30	(0.82)	2.56	(0.88)	0.04

**B_12_ levels**					

B_12_ deficiency (<133 pmol/l) (%)	19		8		<0.01
B_12_ (pmol/l)	337.80	(280.34)	331.24	(247.61)	0.87

**Medications**					

Protein pump inhibitor (%)	45.8		42.9		0.81
Calcium supplements (%)	19.4		10.5		0.09
Multivitamins supplements (%)	14.5		14.5		0.99
Vitamin B supplements (%)	30.6		33.8		0.76
Sulfonylurea (%)	29.4		37.9		0.26

**Neuropathy assessments**					

DPN (%)	39		30		0.21
VPT (V)	14.75	(12.15)	12.22	(9.07)	0.30
Neuropathic pain (%)	33		31		0.91
DN4	3.13	(3.08)	2.89	(2.63)	0.58

*Data are presented as mean SD unless otherwise stated. Unpaired t- and χ^2^ tests were applied to compare continuous and categorical data, respectively between the groups*.

*SBP, systolic blood pressure; DBP, diastolic blood pressure; BMI, Body mass index; DPN, Diabetic peripheral neuropathy; VPT, vibration perception threshold; DN4, neuropathic pain diagnostic questionnaire*.

Of the 362 T2DM patients, 32 (8.8%) fulfilled the criteria for B_12_ deficiency (serum B_12_ <133 pmol/l). However, in those with B_12_ deficiency, the percentage taking Metformin was significantly lower than in those without B_12_ deficiency (60 vs 80%, *P* = 0.03). Patients with B_12_ deficiency were significantly younger (49.16 ± 9.72 vs 54.56 ± 12.71, *P* = 0.01) and had a shorter duration of diabetes (7.03 ± 5.39 vs 11.68 ± 7.89, *P* < 0.001), but comparable SBP, DBP, BMI, HbA1c, cholesterol, triglycerides, HDL, and LDL. The prevalence and severity of neuropathy and neuropathic pain was comparable between those with and without B_12_ deficiency (Table 2).

**Table 2:** Comparison of demographic and clinical characteristics between those with (serum B_12_ <133 pmol/l) and without B_12_ deficiency.

	B_12_ deficiency				
	Yes (*n* = 32)		No (*n* = 330)		*P* value
**Demographics**					

Metformin (%)	60		80		0.03
Age (years)	49.16	(9.72)	54.56	(12.71)	0.01
Diabetes duration (years)	7.03	(5.39)	11.68	(7.89)	<0.001
SBP (mmHg)	127.42	(15.47)	131.22	(19.58)	0.21
DBP (mmHg)	77.93	(11.32)	76.95	(9.72)	0.66
BMI (kg/m^2^)	31.39	(6.48)	32.16	(7.60)	0.55
HbA1c (%)	7.76	(1.97)	7.99	(1.92)	0.52
Total cholesterol (mmol/l)	4.30	(1.04)	4.47	(1.14)	0.39
Triglycerides (mmol/l)	1.53	(0.91)	1.77	(1.07)	0.19
HDL (mmol/l)	1.07	(0.41)	1.08	(0.34)	0.94
LDL (mmol/l)	2.61	(0.84)	2.49	(0.87)	0.49

**Neuropathy assessments**					

DPN (%)	32		33		0.85
VPT (V)	11.87	(9.51)	12.65	(9.19)	0.62
Neuropathic pain (%)	31		32		0.79
DN4	2.47	(2.98)	3.04	(2.59)	0.27

*Data are presented as mean SD. Unpaired t- and χ^2^ tests were used to compare continuous and categorical data, respectively between the groups*.

*SBP, systolic blood pressure; DBP, diastolic blood pressure; BMI, Body mass index; DPN, Diabetic peripheral neuropathy; VPT, vibration perception threshold; DN4, neuropathic pain diagnostic questionnaire*.

### Association Between VPT, DN4 Score, and B_12_

Simple linear regression analysis showed that VPT was positively associated with B_12_ (*r* = 0.18 *P* < 0.001). However, multiple linear regression analysis showed that this association was lost (β = 0.003, *P* = 0.25) after adjustment for confounding factors, including, age, diabetes duration, SBP, HbA1c, and PPI use. DN4 had no association with B_12_ levels.

Simple linear regression analysis shows that B_12_ levels were not associated with the use of Metformin, sulfonylurea, or calcium supplementation, but were associated with age (*r* = 0.15, *P* < 0.01), duration of diabetes (*r* = 0.16, *P* < 0.01), HbA1c (*r* = 0.11, *P* = 0.05), vitamin D (*r* = 0.17, *P* < 0.01), PPI use (*r* = 0.11, *P* < 0.05), multivitamin use (*r* = 0.11, *P* < 0.05), and B_12_ supplementation (*r* = 0.13, *P* < 0.05). However, multiple linear regression analysis showed that B_12_ levels maintained an association only with HbA1c (β = 12.72, *P* = 0.04) and vitamin D use (β = 2.72, *P* = 0.02), after adjustment for confounding factors.

## Discussion

This is the first study from the Middle East region to assess the association between Metformin exposure and B_12_ levels and its relationship to diabetic neuropathy. We show no difference in B_12_ levels between Metformin and non-Metformin users and actually show that the prevalence of B_12_ deficiency was lower in patients on Metformin. This is in contrast to some but not all previously published studies (12). Furthermore, we show no difference in the prevalence of DPN or painful diabetic neuropathy in T2DM patients with and without B_12_ deficiency.

The 2018 ADA Clinical Practice Recommendations endorse screening Metformin users for vitamin B_12_ deficiency and the 2017 ADA diabetic neuropathy statement recommends that all patients with diabetic neuropathy should be assessed for B_12_ deficiency, to exclude a treatable cause of neuropathy (13). However, previous studies examining the relationship between Metformin use and B_12_ deficiency (12); and indeed between B_12_ deficiency and neuropathy have been conflicting (11, 14–18). Indeed, a study has shown a lower prevalence of DPN in T2DM patients on Metformin compared to those not on Metformin (6). Ahmed et al. (17) used the neuropathy total scoring system (NTSS) and showed that subjects with normal B_12_ levels had a comparable prevalence of DPN to those with low B_12_ levels (36.8 vs 32.3%), and no correlation between B_12_ levels and NTSS (17). Russo et al. compared 79 subjects with DPN and 184 without DPN and found no relationship to Metformin use (16). Chen et al. using a neurothesiometer and monofilaments in addition to a structured questionnaire also showed no relationship between Metformin use and peripheral neuropathy (25). In contrast Singh et al. showed that Metformin users had lower levels of B_12_ and a higher Toronto Neuropathy Scoring System (14). Roy et al. showed that patients on Metformin had a lower level of B_12_ and a reduction in median, ulnar, and peroneal nerve conduction (15). In the DPPOS study, while Metformin was associated with an increased risk of B_12_ deficiency, only 13 of the 56 participants on Metformin with low vitamin B_12_ had neuropathy, but there was no difference in neuropathy symptoms or the total Michigan Neuropathy Screening Instrument score (7). A recent study from India has shown an association between Metformin use and B_12_ levels as well as DPN assessed using the Toronto Clinical Scoring System and median, ulnar, peroneal, and posterior tibial nerve conduction velocity (26).

Given that we showed a lower prevalence of B_12_ deficiency in patients taking Metformin, we assessed confounding factors such as other medications, which may alter B_12_ levels. Sulfonylurea use in combination with Metformin is a significant independent risk factor for B_12_ deficiency (8). B_12_ levels have also been reported to be lower in older adults with prolonged PPI and H2 blocker use in one study (27) but not in another study (28). Vitamin B supplementation is prevalent in the Middle East and may also influence B_12_ levels (29, 30). We show no association between B_12_ levels and concomitant use of sulfonylureas or calcium supplementations, but we do show small and significant associations with age, duration of diabetes, HbA1c, and treatment with vitamin D, PPI’s, multivitamins, and B_12_.

This is the first study to assess the relationship between Metformin use B_12_ deficiency and the prevalence and severity of DPN and diabetic painful neuropathy in Qatar. The prevalence of both sensory neuropathy and painful diabetic neuropathy was comparable to previously published data (31–33). A limitation of this study is that it is a retrospective cohort study, but VPT and DN4 were assessed without the investigators being aware of the treatment or B_12_ status. The majority of patients had been prescribed Metformin as first-line therapy in accord with international guidelines, unless they were intolerant or it was withdrawn (34), and, therefore, it was not possible to recruit a larger number of patients not on Metformin. We cannot establish the exact duration of Metformin exposure, although we can assume that Metformin was prescribed shortly after diagnosis and, therefore, exposure is approximately equivalent to the duration of diabetes, which was approximately 10 years. As noted in the meta-analysis of Chapman et al. the B_12_ lowering effect of Metformin occurs within 6 weeks to 3 months of commencing Metformin (12).

In conclusion, we show no difference in B_12_ levels or the severity of DPN or painful diabetic neuropathy in Metformin compared to non-Metformin users. We also show no difference in vibration perception or painful diabetic neuropathy in those with and without B_12_ deficiency. These data urge the need for further larger, prospective studies to confirm or refute the current findings to support or challenge the highly prevalent practice of prescribing B_12_ for neuropathy across the Middle East.

## Ethics Statement

This study was carried out in accordance with the recommendations of Investigator guidance: Investigator obligations (HRP-800), Institutional Review Board (IRB) of WCM-Q and HMC. The protocol was approved by the IRB of WCM-Q and HMC. All subjects gave written informed consent in accordance with the Declaration of Helsinki.

## Author Contributions

RM, TE, and GP had full access to all the data in the study and take responsibility for the integrity of the data and the accuracy of the data analysis. Study concept and design: RM and TE. Acquisition, analysis, or interpretation of data: all authors. Drafting of the manuscript: RM, TE, and GP. Critical revision of the manuscript for important intellectual content: all authors. Statistical analysis: GP and RM. Administrative, technical, or material support: all authors.

## Conflict of Interest Statement

The authors declare that the research was conducted in the absence of any commercial or financial relationships that could be construed as a potential conflict of interest.
